# Using a Twitter Chat to Rapidly Identify Barriers and Policy Solutions for Metastatic Breast Cancer Care: Qualitative Study

**DOI:** 10.2196/23178

**Published:** 2021-01-15

**Authors:** Riti Shimkhada, Deanna Attai, AJ Scheitler, Susan Babey, Beth Glenn, Ninez Ponce

**Affiliations:** 1 Center for Health Policy Research University of California, Los Angeles Los Angeles, CA United States; 2 Geffen School of Medicine University of California, Los Angeles Los Angeles, CA United States; 3 Fielding School of Public Health University of California, Los Angeles Los Angeles, CA United States

**Keywords:** metastatic breast cancer, Twitter, infodemiology, infoveillance, health care barriers, health care policy, social media, policy, breast cancer

## Abstract

**Background:**

Real-time, rapid assessment of barriers to care experienced by patients can be used to inform relevant health care legislation. In recent years, online communities have become a source of support for patients as well as a vehicle for discussion and collaboration among patients, clinicians, advocates, and researchers. The Breast Cancer Social Media (#BCSM) community has hosted weekly Twitter chats since 2011. Topics vary each week, and chats draw a diverse group of participants. Partnering with the #BCSM community, we used Twitter to gather data on barriers to care for patients with metastatic breast cancer and potential policy solutions. Metastatic breast cancer survival rates are low and in large part conditioned by time-sensitive access to care factors that might be improved through policy changes.

**Objective:**

This study was part of an assessment of the barriers to care for metastatic breast cancer with the goal of offering policy solutions for the legislative session in California.

**Methods:**

We provided 5 questions for a chat specific to metastatic breast cancer care barriers and potential policy solutions. These were discussed during the course of a #BCSM chat on November 18, 2019. We used Symplur (Symplur LLC) analytics to generate a transcript of tweets and a profile of participants. Responses to the questions are presented in this paper.

**Results:**

There were 288 tweets from 42 users, generating 2.1 million impressions during the 1-hour chat. Participants included 23 patient advocates (most of whom were patients themselves), 7 doctors, 6 researchers or academics, 3 health care providers (2 nurses, 1 clinical psychologist), and 2 advocacy organizations. Participants noted communication gaps between patient and provider especially as related to the need for individualized medication dosing to minimize side effects and maximize quality of life. Timeliness of insurance company response, for example, to authorize treatments, was also a concern. Chat participants noted that palliative care is not well integrated into metastatic breast cancer care and that insurance company denials of coverage for these services were common. Regarding financial challenges, chat participants mentioned unexpected copays, changes in insurance drug formularies that made it difficult to anticipate drug costs, and limits on the number of physical therapy visits covered by insurance. Last, on the topic of disability benefits, participants expressed frustration about how to access disability benefits. When prompted for input regarding what health system and policy changes are necessary, participants suggested a number of ideas, including expanding the availability of nurse navigation for metastatic breast cancer, developing and offering a guide for the range of treatment and support resources patients with metastatic breast cancer, and improving access to clinical trials.

**Conclusions:**

Rapid assessments drawing from online community insights may be a critical source of data that can be used to ensure more responsive policy action to improve patient care.

## Introduction

Web-based social media platforms have changed the face of support networks, breaking down the barriers of distance, time, and physical limitations to bring together diverse voices in a common cause. For social scientists interested in hearing current conversations from social networks, these platforms offer access to a sample of engaged key informants and a way to generate rapid insights. Twitter has been increasingly used to carry out public health research such monitoring diseases and outbreaks, gauging public opinion to emerging health threats, and implementing health education campaigns [1-6]. Prior work suggests that Twitter may aid policy-makers engage their constituents beyond what is possible through in-person meetings or passive communication (eg, listservs, newsletters) [4,5,7-9]. A growing number of patients use Twitter to connect with their peers as well as clinicians and researchers for information, psychosocial support, and research and advocacy opportunities; patients treated for breast cancer are one of the most engaged groups on social media platforms for this purpose [1,10-15].

Breast cancer is the most commonly diagnosed cancer among women in the United States, with approximately 276,000 women diagnosed each year [16]. Metastatic breast cancer, or stage 4 breast cancer, occurs when breast cancer spreads to other sites, most commonly the lungs, liver, bones, and brain. Patients with metastatic breast cancer are usually on some form of treatment for their remaining lifespan, which may include hormone blockade, targeted therapy, chemotherapy, immunotherapy, surgery, or radiation therapy. While there have been improvements in treatment options available to individuals with metastatic breast cancer, there is no cure and every year, in the United States, approximately 42,000 women will die from breast cancer [16]. Because of the intensity of the disease and its treatment, patients with metastatic breast cancer must stay engaged in the health care system and in close contact with their treatment team to receive timely care tailored to their specific needs and preferences.

The Twitter Breast Cancer Social Media (#BCSM) community was cofounded by two breast cancer patient advocates in 2011 and is the first and longest-running cancer support community on Twitter. The #BCSM community hosts weekly tweet chats (live Twitter events) using the #BCSM hashtag to provide information and virtual support to all impacted by breast cancer. Topics vary each week, and a diverse community of patients (eg, men and women, early and late stage disease), doctors and other health care providers, researchers, and representatives from advocacy organizations participate [1]. Recognizing that online patient communities can be a valuable source of timely, real-world data [17,18], which can guide policy change, the study team partnered with the #BCSM community to host a Twitter chat to gather data on barriers to care and potential policy solutions for patients living with metastatic breast cancer. Barriers to care are factors that impede or limit a patient’s access to health care services [19,20]. The study aimed to collect information on the experiences of patients living with metastatic breast cancer, health care providers, advocates, and support service providers who work with patients with metastatic breast cancer. This study was part of a larger initiative to gather stakeholder input in informing policy proposals to improve care for women with metastatic breast cancer for the next California legislative session, thus the timeliness of data gathering was critical. Our objective was to assemble a list of priority areas and policy recommendations to improve metastatic breast cancer care from chat participants on Twitter who have been highly engaged in breast cancer care advocacy, research, and care delivery. These recommendations will be included in a report intended for policy makers who are looking for ways to improve metastatic breast cancer care.

## Methods

The University of California, Los Angeles institutional review board determined that the study was exempt from review due to the public nature of conversations held on Twitter. The study team designed 5 questions to solicit input about metastatic breast cancer care barriers and potential policy solutions. These questions were informed by prior research on barriers to care for women with earlier stage breast cancer [21], a literature review conducted for this study, and feedback from key stakeholders on metastatic breast cancer. We worked with #BCSM comoderator (since 2011) and study partner (DJA), to identify the ideal number of questions and wording for the chat questions so that questions could be asked within a 1-hour timeframe.

The questions were posted on November 13, 2019 on a breast cancer information blog maintained by author DJA that was shared on Twitter with the #BCSM tag to prepare and inform likely participants of the scheduled 1-hour Twitter chat on November 18, 2019. The questions were (1) What are some of the most significant health care communication barriers faced by patients with metastatic breast cancer? (2) What are the palliative care barriers faced by those with metastatic breast cancer? (3) What are the financial challenges faced by patients w with metastatic breast cancer? (4) What are barriers to obtaining disability? (5) What health system or policy changes would you suggest to improve the care experience for patients with metastatic breast cancer?

During the introduction portion of the chat, participants were informed by the moderator (DJA) that their tweets would be used for a research study, and those who did not want to participate in the study should not tweet using the #BCSM hashtag for the 1-hour duration of the scheduled chat. This study’s principal investigator (NP) was introduced at the start of the chat and was an active participant in the discussion. During the chat, the moderator posted the 5 questions sequentially and allowed for conversation and questions, per usual tweet chat routine.

Publicly available information from Symplur (Symplur LLC), a health care focused Twitter database and analytics company, was used to generate a transcript of tweets from the 1-hour chat. Only tweets that were in direct responses to each of the posed questions (ie, no retweets or off-topic entries) were evaluated. All direct and unique responses to each question were compiled for presentation in the paper by one author (RS) and checked by another author (AS). Symplur Signals (Symplur LLC) was used to categorize participants into the following groups: doctor, health care provider (not doctor), patient advocate, advocacy organization, health care organization, or research/academic. For health care stakeholders classified in more than one category, a manual review of Twitter profiles was performed to determine the category in which the participant best fit. We include the category of each participant for each Twitter chat response to help readers understand the perspective or experience of each participant.

## Results

During the course of the 1-hour chat, there were 288 tweets from 42 unique participants. This generated 2.1 million impressions (a measure of tweet reach, indicating potential views). Participants included 23 patient advocates (most of whom were further identified as patients with breast cancer based on their Twitter profiles), 7 doctors, 7 researchers/academics, 3 health care providers (2 nurses, 1 clinical psychologist), and 2 representatives of advocacy organizations (Table 1). Based on their Twitter biographies, participants resided in the United States (n=40), Canadian (n=1), and unknown (n=1). More granular geolocation data were not available for all of the participants to further identify each participant’s city or state of residence.

Representative tweets generated by the participants in response to each of the questions are shown in Table 2, and full results of the Twitter chat are shown in Multimedia Appendix 1. The first question inquiring about communication barriers to care generated numerous responses such as communication gaps between patient and provider, especially regarding communicating disease progression and quality of life. Timeliness of insurance company response, for example, to authorize treatments, was also a concern.

The second question focused on palliative care, which includes the management of the side effects of treatments and treatment of the symptoms of disease to optimize quality of life. Palliative care includes a number of domains of care: physical, social, cultural, emotional, spiritual, structural, psychological, and end of life [22]. In their responses, chat participants noted that palliative care is not well integrated into metastatic breast cancer care and that insurance company denials of coverage for these services were common. Regarding financial challenges, chat participants mentioned unexpected copays, changes in insurance drug formularies that made it difficult to anticipate drug costs year to year, and limits on the number of physical therapy visits covered by insurance. On the topic of disability benefits, participants expressed frustration that there is a lack of clear guidance available on how to access disability benefits. When prompted for input regarding needed health system and policy changes, participants suggested expanding the availability of nurse navigation for metastatic breast cancer, developing a guide for the range of treatment and support resources for patients at the time of metastatic breast cancer diagnosis, and improving access to clinical trials, specifically, reimbursing the cost of travel and accommodations to and from the trial site.

**Table 1:** Description of #BCSM community Twitter chat participants.

Participant type	Participants (N=42), n (%)	Tweets, n (%)
Patient or advocate	23 (55)	129 (45)
Doctor^a,b^	7 (17)	125 (43)
Researcher/academic	7 (17)	18 (6)
Health care provider	3 (7)	6 (2)
Advocacy organization	2 (5)	10 (4)
Total	42 (100)	288 (100)

^a^Includes #BCSM chat moderator (n=99 tweets).

^b^1 participant classified as a doctor is a practicing doctor and a patient with breast cancer in remission.

**Table 2:** Questions and representative responses from participants regarding barriers to care for metastatic breast cancer and potential policy solutions.

Question	Participant tweet
What are some of the most significant health care communication barriers faced by patients with metastatic breast cancer?	*MBC pts often try to look as good as possible at their onc appts and may not report QOL issues unless oncologist asks. Some don’t ask.* [Patient advocate] *Big communication issue is communication style – pt usually has a preference for how detailed they want their [oncologist]. to be. Need to discuss this up front. Otherwise pt often seeks new onc.* [Patient advocate] *I have family members who have been diagnosed with early stage breast cancer and they were unaware that ~20-30% of early stage breast cancers will become MBC. I think this may be a communication gap to patients* [Patient advocate]
What are the palliative care barriers faced by those with metastatic breast cancer?	*Palliative care is not always discussed with patients and not explained well. For a long time I thought palliative care and hospice were synonymous.* [Patient advocate] *My plan’s Palliative Care Team is still figuring out what they do for a living. Right now focus is mainly advanced directives and pain meds [medications]. Should get better but not yet.* [Patient advocate] *A hospital or cancer center having onsite palliative care or ability to have access to Palliative Care is a huge issue for countless patients. Many are referred to pain clinics which are NOT the same at all. There is an alarming shortage of/access to Palliative Care.* [Patient advocate]
What are the financial challenges faced by patients with metastatic breast cancer?	*Surprise co-pays for new therapies once the line of therapy is established. Then scrambling for payment assistance when already stressed by progression.* [Patient advocate] *Was recently told that I could not continue PT [physical therapy] because I had already had 35 and anything beyond would be out of pocket. So now I either pay for PT or just wait until January.* [Patient advocate] *To make matters worse/more frustrating - for treatment meds taken at home... Many private insurers change their formulary lists twice/year…MD [doctor] offices often can't keep up with those changes & pts find out after the fact.* [Health care provider]
What are barriers to obtaining disability?	*Big barrier to getting disability is the pt doesn’t know the process of applying if employed, small companies don’t know what to do.* [Patient advocate] *There are lots of people who are contract workers (especially in high tech). They don’t have disability insurance as aren’t aware of state disability.* [Patient advocate] *A lot of times pts have to be off of work consecutively for 12 weeks before their application is looked at. And you have to exhaust all of your benefit time as well.* [Advocacy organization]
What health system or policy changes would you suggest to improve the care experience for patients with metastatic breast cancer?	*Besides patient centered dosing, we need to make it easier to be in clinical trials without requiring expensive travel costs to pts. We limit who can be in a trial by pts who are unable to pay for travel.* [Patient advocate] *Would love for [insurance] companies to have a separate group just for metastatic (maybe unrealistic) but would have training and understand the unique needs of that group.* [Patient advocate] *Maybe it would be a good idea to have a national nurse navigator organization that would work like a hotline. So even remote access.* [Patient advocate]

## Discussion

In partnership with an online breast cancer community, we identified several areas where legislation, policy change, or greater investment of resources can be made to improve metastatic breast cancer care. Many of the barriers to care relate to communication, care coordination, and insurance authorization. This study was part of an assessment of the barriers to care for metastatic breast cancer with the goal of offering policy solutions for the legislative session in California.

Chat participants engaged in conversations around communication barriers, echoing the need to lift communication and care coordination barriers from the patient-provider relationship [23]. Chat participants noted that patients with metastatic breast cancer, compared to patients with nonmetastatic disease have treatment decision preferences that focus far more on quality of life [24,25], and may benefit from protocols that require routine reporting of quality of life to providers. Furthermore, improved access to nurse navigation for patients with metastatic cancer could reduce the burden of care coordination on the patient and their caregiver.

Chat participants noted a number of difficulties with insurance approval of treatments. Step therapy or a fail-first protocol is an insurer's policy that requires a patient to try therapies in a specific order (ie, try a less expensive generic or biosimilar version of a therapy before moving up a step to the more expensive therapy). These processes could impose barriers to access and delays in receiving the most effective treatment [26]. For patients with metastatic breast cancer who face a 5-year survival rate of only 27% [16], fail-first protocols are especially penalizing, protracting access to a drug that may be preferred by the patient and covered by the patient’s insurer, thus dually harming the health and financial well-being of women with metastatic breast cancer. There are currently 8 states (Arizona, Colorado, Illinois, Louisiana, Maryland, Minnesota, North Dakota, and Texas) that have laws restricting the use of step therapy or prior authorization protocols for patients with stage 4 or metastatic cancer; other states are considering similar legislation [27].

Even when coverage for a particular treatment is approved by insurance, cost of the medication or service remains a concern as the patient is responsible for copayment for the treatment set forth by the plan design. Financial concerns and barriers were expressed by patients in our chat. Given that many metastatic breast cancer medications are specialty or in the tie of high-cost drugs, patients are responsible for a greater share of the cost compared to that for nonspecialty drugs. This is particularly a burden for patients who are covered by high-deductible health plans [28].

Disease progression is one of several patient factors associated with financial distress [29], and patients with metastatic breast cancer may wish to address costs at the time of treatment decisions [30]. The American Society of Clinical Oncology encourages cost discussions between patients and providers [31]. The increased focus on value-based care as well as attention to financial toxicity experienced by patients undergoing treatment for cancer has made it even more important that clinicians take on a role of financial advocate for their patients, although this is an area where physicians do not always feel comfortable [32-35]. There are calls for more incentives for cost discussions and subsequent reduction in financial burden among patients using the personal spending burden (measured as personal expenditures for health care relative to income) as a quality-of-care metric [36] and calls to enact reform that makes cost transparent to patients in the prior authorization process [37]. Other efforts to lower costs to patients include the enactment of oral chemotherapy parity laws to limit patient out-of-pocket costs in line with intravenous administered drugs. For example, in 2018 California Assembly Bill 1860 [38] capped patients’ out-of-pocket costs for oral chemotherapy to US $250/month per drug. A federal bill, the Cancer Drug Parity Act [39], seeks to bring parity in oral and intravenous chemotherapy to all states in the US.

Tweet chat participants noted that palliative care was often not discussed or there were restrictions regarding participation. California improved access to palliative care with the introduction of Senate Bill 1004 [40] in 2014, which required the California Department of Health Care Services to expand community-based palliative care services to its Medicaid beneficiaries. However, while the bill was a major step forward for patients with advanced diseases such as metastatic breast cancer in terms of access to palliative care, patients may be underusing these services due to lack of referral or appropriate care coordination by their oncologist and misconceptions regarding palliative care versus hospice or end-of-life care. In addition, there may be potential language and cultural barriers [41,42].

Barriers to accessing disability benefits also emerged during the chat. The federal 2019 Metastatic Breast Cancer Access to Care Act [43] would waive the current waiting periods for federal disability benefits of 5 months for Social Security disability benefits and 2 years for Medicare for those younger than 65 years. While the bill, if enacted, would lift the wait to access benefits, tweet chat participants mentioned a need for improved awareness in applying for disability programs and knowing what is available to them. Patient navigation and support communities may offer opportunities for improving literacy in accessing benefits and financial support [44].

Metastatic breast cancer advocates have continued to press for meaningful changes in policy to improve care through virtual lobbying and awareness efforts. Decisions regarding the Metastatic Breast Cancer Access to Care Act [43] and the Cancer Drug Parity Act [39] had still not been made (as of the time this paper went to press). While these two federal bills are important steps forward, state mandates and local health system policy shifts may be able to bring consequential changes to access and costs through changes in prior authorization practices and support programs.

Our analysis has several limitations. The participants in the chat were not randomly selected; those who took part in the discussions were likely those most familiar with Twitter and the weekly #BCSM chats and adept at the chat format. We do not know the geographic location, age, or disease severity of the participants. Participants in online breast cancer communities may not be representative of the average patient population [45]. We only used one particular hashtag, #BCSM, to link our tweets during the chat; there are other hashtags related to metastatic breast cancer that we did not include but might have improved engagement. Stakeholder characterization was based on self-reported information (in a Twitter user’s biography) and a proprietary (Symplur LLC) algorithm. Further work is needed to determine how to best utilize the information discussed by patients on various social media platforms to thoughtfully inform public policy decisions. Despite these limitations, our findings suggest that Twitter can be an important source of timely data on the struggles and barriers being faced by patients with cancer and other health conditions.

This Twitter chat elicited a number of policy or program ideas that may improve barriers to care for patients with metastatic breast cancer. Multiple participants reported the lack of patient navigation for metastatic breast cancer and felt a policy priority could be to initiate a navigation program, hotline, or guide for all services available to a patient undergoing treatment. One participant felt metastatic cancers are so different from nonmetastatic cancers that there might be a need for physician groups who treat just metastatic cancers. Another participant mentioned clinical trial access might be improved if travel costs to and from trial sites were covered. Participants noted the need for policies related to improving palliative care and better quality-of-life reporting. Participants also noted financial and insurance barriers that might be addressable through health mandates, such as restricting formulary switching so that medications remain covered by insurance once a patient starts the treatment, limiting restrictions on number of physical therapy visits, and limiting surprise copayments. All recommendations brought up by #BCSM Twitter chat participants are included in a report that will be disseminated to policy makers working to improve timely access to care for women living with metastatic breast cancer in California.

Rapid assessments drawing from various online patient communities, not just those focused on cancer, may provide critical, timely information that can be used to ensure more responsive policy action. This has become even more important as the health care system adjusts in response to the COVID-19 pandemic.

## Appendix

Multimedia Appendix 1 Table S1. Full list of Twitter chat responses from participants regarding barriers to care for metastatic breast cancer and potential policy solutions.

## Abbreviations

#BCSM: Breast Cancer Social Media community
COVID-19: coronavirus disease 2019
