# Supplementary material for: Using a Twitter Chat to Rapidly Identify Barriers and Policy Solutions for Metastatic Breast Cancer Care: Qualitative Study
Source: JMIR Public Health Surveill. 2021 Jan 15;7(1):e23178. doi: 10.2196/23178 (PMC7872835; doi:10.2196/23178)
Supplement: Multimedia Appendix 1 [file publichealth_v7i1e23178_app1.docx]

Supplemental Table 1. **Full list of Twitter chat responses from participants regarding barriers to care for MBC and potential policy solutions**

| Question | Participant Tweet |
| --- | --- |
| Q1 - what are some of the most significant healthcare communication barriers faced by patients w/metastatic breast cancer? | “One big issue is agreeing on next line of therapy when it’s chemo esp [especially] when onc [oncologist] insists on giving maximum dose. Just spoke to helpline caller today & she was freaking out about being able to have QOL [quality of life] with new tx [treatment] suggested. Need pt [patient] centered dosing.” – Patient advocate  “MBC pts often try to look as good as possible at their onc appts and may not report QOL issues unless oncologist asks. Some don’t ask.” – Patient advocate  “MBC is not treated under a curative manner like early stage. Goal is to extend length and QOL. Lower doses have been shown to be effective. So why throw an atomic bomb at an MBC pt who obviously won’t tolerate it?” – Patient advocate  “From an insurance perspective, they don't always feel the urgency. Just waiting on approvals can feel like an eternity when there is progression” – Patient advocate  “What about insurance pre-approvals for routine scans and being rejected? That is a real anxiety builder for MBC pts who are vulnerable because they don’t know if they’ve had progression. Sleepless nights.” – Patient advocate  “As I reflect back on the "chaos" of #metastatic #breastcancer faced by #theloveofmylife obviously, I think about Maureen and her pain first. But that same "chaos" is external... docs, insurance, pharmacy, etc.” – Patient advocate  “There are misunderstandings in terminology. Onc says “Your MBC is stable” and [patient] hears “your tumors aren’t shrinking. Bad news!”” – Patient advocate  “Words do matter & need to be operationally defined & translated from med-speak to pt-speak With many chronic med conditions, "stable" & "no change" are good/great, yet freq misinterpreted by pts asking the pt what they heard helps” – Patient advocate  “Big communication issue is communication style – pt usually has a preference for how detailed they want their [oncologist] to be. Need to discuss this up front. Otherwise pt often seeks new onc.” – Patient advocate  “Ppl presenting with unusual symptoms that end up being dx’d as MBC are often missed & dismissed prolonging time to treatment. “If the doc hasn’t seen it before, it doesn’t exist” (Eg young age, etc)” – Patient advocate  “I have family members who have been diagnosed with early stage breast cancer and they were unaware that ~20-30% of early stage breast cancers will become MBC. I think this may be a communication gap to patients” – Patient advocate  “It is so important to let patients know that they need to be aware of disease progression and that there are symptoms they should be cognizant of.” – Patient advocate  “Onc needs to verify - ask what the patient understands - to find out if the patient is able to listen and process info. I spend time w MBC helpline callers making sense of what caller thought they heard from onc” – Patient advocate |
| Q2 - what are the palliative care barriers faced by those with metastatic breast cancer? | “I heard earlier from pt on clinical trial, couldn't keep palliative care team” - Doctor  “Palliative care is not always discussed with patients and not explained well. For a long time I thought palliative care and hospice were synonymous.” – Patient advocate  “Such a common misconception! [referring to palliative care being synonymous with hospice] Think of palliative care as symptom management. Can be appropriate for ANY stage of cancer” – Patient advocate  “My plan’s Palliative Care Team is still figuring out what they do for a living. Right now focus is mainly advanced directives and pain meds [medications]. Should get better but not yet.” – Patient advocate  “A hospital or cancer center having onsite palliative care or ability to have access to Palliative Care is a huge issue for countless patients. Many are referred to pain clinics which are NOT the same at all. There is an alarming shortage of/access to Palliative Care.” – Patient advocate  “Denials from insurance companies for meds/treatments that have been proven standard of care & deemed necessary by patients oncologist. Fear of step therapy. Fear of FDA limiting access to needed/necessary drugs to function and have decent QOL.” – Patient advocate  “After waiting for authorization, then have to wait for scheduling. can be a very long process especially once you have been told about progression.” – Patient advocate  “Just had two major hospital systems in my local area both lose their top #PalliativeCare experts. Devastating loss to the patient communities. We need more than 1 expert at a hospital to serve all patients.” – Patient advocate |
| Q3 - what are the financial challenges faced by patients w/metastatic breast cancer? | “Surprise co-pays for new therapies once the line of therapy is established. Then scrambling for payment assistance when already stressed by progression.” – Patient advocate  “Was recently told that I could not continue PT [physical therapy] because I had already had 35 and anything beyond would be out of pocket. So now I either pay for PT or just wait until January.” – Patient advocate  “To make matters worse/more frustrating - for treatment meds taken at home... Many private insurers change their formulary lists twice/year…MD [doctor] offices often can't keep up with those changes & pts find out after the fact.” – Health care provider  “Disability. I haven't been down this road, but know people who have. Stressful when [it’s] reviewed and you have to go [through] whole process again.” – Patient advocate  “Cost of meds for sure and access to care. Healthcare teams could probably do a better job of finding resources for pts and making sure they are aware of possible grants, manufacture coupons etc”- Advocacy organization  “What I've heard from others that there are not nearly enough #PalliativeCare teams, and the ones in place are under-utilized” – Doctor |
| Q4 – what are barriers to obtaining disability? | “Big barrier to getting disability is the pt doesn’t know the process of applying if employed, small companies don’t know what to do.” – Patient advocate  “[Regarding] disability I've also seen some tweets from patients that if they apply for medication financial assistance program that can limit their disability... doesn't seem right” - Doctor  “There are lots of people who are contract workers (especially in high tech). They don’t have disability insurance as aren’t aware of state disability.” – Patient advocate  “I’m a doc and a patient, Stage 2 2015, currently NED [no evidence of disease]. I have been on and off disability, complete and partial at times. Hard to meet criteria, especially when reason is combo of disabilities/problems.” – Doctor  “A lot of times pts have to be off of work consecutively for 12 weeks before their application is looked at. And you have to exhaust all of your benefit time as well.” – Advocacy organization |
| Q5 – what health system or policy changes would you suggest to improve the care experience for patients w/metastatic breast cancer | “Besides patient centered dosing, we need to make it easier to be in clinical trials without requiring expensive travel costs to pts. We limit who can be in a trial by pts who are unable to pay for travel.” – Patient advocate  “Would love for [insurance] companies to have a separate group just for metastatic (maybe unrealistic) but would have training and understand the unique needs of that group.” – Patient advocate  “We need MBC pts to undergo retesting for receptor status & genetic changes upon progression, esp when pt was on a therapy for a good amount of time. If we don’t, next therapy line may be totally ineffective.” – Patient advocate  “Maybe it would be a good idea to have a national nurse navigator organization that would work like a hotline. So even remote access.” – Patient advocate  “Obviously we need resources. We need support, we can’t find things we don’t know exist. There should be a universal guide given to all patients at diagnosis.” – Patient advocate  “Metastatic patients don’t have nurse navigators.” – Patient advocate  “Nurse navigators aren’t always diagnosis specific, they are clinic & practice specific (or they freelance). Sadly, they aren’t widely available. Some cancer ctrs [centers] have none, some have several, & some private oncology groups have 2! Very uneven.” – Patient advocate  “I’m too tired to type now but have list of challenges. Constant battle to get care from health teams that will preserve QOL. Neuropathy, GERD, rare side effects, and on and on. I’m adept at article reviews to take info to doc. But it’s exhausting.” – Patient advocate  “There’s so much information for one to process. Maybe having someone from the care team following up with the patient a few days later would be helpful.” – Advocacy organization  “How about oncologists who treat only #metastaticcancer patients so they can focus. And also, how about someone at each hospital who scans literature for latest and updates medical teams as a job.” – Patient advocate  “I became a pt advisor thru our cancer control division of healthcare and an/ peer support person thru local cancer support center. We need more nonmedical cancer support centers.” – Patient advocate  “I think main point is #metastaticcancer is world of its own. Dose reduce. Keep QOL in forefront. Offer emotional & financial resources. Call it Supportive Care. Offer assistance in researching. Coordinate with other specialties. Ease access to records.” – Patient advocate  “This is what my area needs - nurse-navigators. And advocates. Even teleadvocates.” - Doctor |
